# Prevalence and Risk Factors of Mental Health Problems Among Healthcare Workers During the COVID-19 Pandemic: A Systematic Review and Meta-Analysis

**DOI:** 10.3389/fpsyt.2021.567381

**Published:** 2021-06-15

**Authors:** Qinjian Hao, Dahai Wang, Min Xie, Yiguo Tang, Yikai Dou, Ling Zhu, Yulu Wu, Minhan Dai, Hongmei Wu, Qiang Wang

**Affiliations:** ^1^The Center of Gerontology and Geriatrics, West China Hospital of Sichuan University, Chengdu, China; ^2^Department of Psychiatry, Hospital of Chengdu Office of People's Government of Tibetan Autonomous Region, Chengdu, China; ^3^Mental Health Center and Psychiatric Laboratory, West China Hospital of Sichuan University, Chengdu, China

**Keywords:** coronavirus disease, healthcare workers, mental health, prevalence, risk factors, meta-analysis

## Abstract

**Objective:** The purpose of this meta-analysis was to summarize the prevalence and risk factors of mental health problems among healthcare workers during the COVID-19 pandemic.

**Methods:** We applied an optimized search strategy across the PubMed, EMBASE, Scopus, PsycINFO, and four Chinese databases, with hand searching supplemented to identify relevant surveys. Studies were eligible for inclusion if they were published in peer-reviewed literature and used a validated method to assess the prevalence and risk factors of mental health problems among healthcare workers during the COVID-19 pandemic. Heterogeneity was quantified using *Q* statistics and the *I*^2^ statistics. The potential causes of heterogeneity were investigated using subgroup analysis and meta-regression analysis. Sensitivity analysis was performed to examine the robustness of the results.

**Results:** We pooled and analyzed data from 20 studies comprising 10,886 healthcare workers. The prevalence of depression, anxiety, insomnia, post-traumatic stress symptoms, phobia, obsessive–compulsive symptoms, and somatization symptoms was 24.1, 28.6, 44.1, 25.6, 35.0, 16.2, and 10.7%, respectively. Female and nurses had a high prevalence of depression and anxiety. Frontline healthcare workers had a higher prevalence of anxiety and a lower prevalence of depression than the those in the second-line. Furthermore, the proportion of moderate–severe depression and anxiety is higher in the frontline. Additionally, four studies reported on risk factors of mental health problems.

**Conclusions:** In this systematic review, healthcare workers have a relatively high prevalence of depression, anxiety, insomnia, post-traumatic stress symptoms, phobia, obsessive–compulsive symptoms, and somatization symptoms during the COVID-19 pandemic, and focus should be on the healthcare workers at high risk of mental problems. Mental health problems in healthcare workers should be taken seriously, and timely screening and appropriate intervention for the high-risk group are highly recommended.

**Systematic Review Registration:**https://www.crd.york.ac.uk/prospero/display_record.php?ID=CRD42020179189.

## Introduction

At the end of 2019, an emerging infectious disease named coronavirus disease 2019 (COVID-19) caused by severe acute respiratory syndrome coronavirus 2 (SARS-CoV-2) broke out and caused a global pandemic that put healthcare workers (HCWs) across the world under unprecedented challenges and huge psychological impact (1, 2). In the fight against COVID-19, HCWs played a leading role. The HCWs were in the vanguard of the battle to combat COVID-19, providing medical services to the most affected areas (3). The mental health of HCWs was greatly challenged during the Middle East respiratory syndrome (MERS) and severe acute respiratory syndrome (SARS) (4–7). As generally known, COVID-19 is more contagious than SARS and MERS (8, 9) and can bring HCWs on the frontline mental health problems (10–14). Similarly, during the COVID-19 pandemic, HCWs encountered a huge psychological burden, with a high prevalence of depression, anxiety, insomnia, and distress (15, 16). Moreover, frontline HCWs, in fighting COVID-19, have more severe degrees of mental health symptoms than other HCWs (15, 17). Beyond the effects of mental health problems on individuals, the mental health problems of HCWs may link to poor-quality patient care and increased medical errors (18, 19). A reliable estimate of the prevalence of mental health problems among HCWs during the COVID-19 pandemic is of vital importance to its prevention, identification, and treatment. To the best of our knowledge, there has not been a meta-analysis of the prevalence of mental health problems and risk factors among HCWs during the COVID-19 pandemic published in the literature. We conducted a systematic review and meta-analysis of the prevalence of mental health problems and risk factors among HCWs during the COVID-19 pandemic to identify at-risk HCWs and provide timely assistance and intervention.

## Methods

### Protocol

The protocol of our study has been registered on the International Prospective Register of Systematic Reviews (PROSPERO, CRD42020179189). The review methods are described in accordance with the Preferred Reporting Items for Systematic Reviews and Meta-analyses guidelines (20) and the Meta-analysis of Observational Studies in Epidemiology criteria (21).

### Search Strategy and Study Eligibility

The search was performed in all fields in the PubMed, EMBASE, Scopus, PsycINFO, and four Chinese databases, including Chinese Biomedical Literature Database, China National Knowledge Infrastructure, China Science and Technology Journal Database, and Wanfang database, with no language restrictions, from January 1, 2020 (subsequent to the emergence of COVID-19 in China) to April 14, 2020. The detailed search terms and full strategies are available in Supplementary Material 1. Additionally, a manual search was performed by reviewing the reference lists of the related articles by two investigators. Where necessary, we contacted the authors for any additional data.

Population-based or hospital-based studies fulfilling the following criteria were included in the present analysis: (1) the HCWs including doctors, nurses, and other medical personnel who were directly or indirectly involved in the diagnosis, treatment, or care of patients with confirmed or suspected cases of COVID-19, (2) studies reported the prevalence or risk factors of mental health problems (depression, anxiety, insomnia, etc.) among HCWs which were assessed by structured interviews or validated questionnaires, (3) cross-sectional or cohort studies, and (4) published in a peer-reviewed journal.

Studies without original data and studies in which the data could not be reliably extracted after corresponding with the authors were excluded. If the same sample was reported in more than one study, the larger sample size with the longest follow-up duration will be included.

### Data Extraction and Quality Assessment

Two authors independently extracted the data, reported by the selected articles, and documented the following details in a standardized table: general information of publication (first author, year and location of the study, study period, and language), study design (cohort or cross-sectional), survey method, sampling method, study sample origin (population-based or hospital-based), sample size, number of HCWs, number of female HCWs, number of mental health problems among HCWs, instrument used to assess mental health problems, risk factors of each mental health problem, and the effects of each risk factor. The methodological quality of the included cross-sectional studies was assessed using an 11-item checklist which was recommended by the Agency for Healthcare Research and Quality (AHRQ). The answer to each item is “no,” “unclear,” and “yes,” respectively. Study quality was defined as follows: low quality (0–3 yes), moderate quality (4–7 yes), and high quality (8–11 yes). Any discrepancies will be resolved by consensus, and if necessary, a third reviewer will be consulted to arbitrate.

### Data Synthesis and Analysis

The pooled prevalence of each mental health problem and its 95% confidence intervals (CI) were calculated using random-effects meta-analysis that accounted for the heterogeneity of studies (22). The heterogeneity of studies was assessed by *Q*-test and *I*^2^. *I*^2^ > 50% and *p* < 0.05 in the *Q*-test were interpreted as the presence of significant heterogeneity (23, 24). When significant heterogeneity was identified, the source of heterogeneity was explored by subgroup analysis and meta-regression. Subgroup analyses were conducted with stratification by sample size, staff type, position, and gender. Sensitivity analysis using the leave-one-out method was performed to examine the robustness of the results. The potential publication bias was evaluated by funnel plot and the Egger linear regression test (25, 26). The statistical tests were two-sided and used a significance threshold of *P* < 0.05. We performed the statistical analysis in R (version 4.0.0; https://www.r-project.org/). A systematic narrative synthesis will be conducted if it is impossible to handle any meta-analysis.

## Results

### Study Characteristics

**Figure 1:**
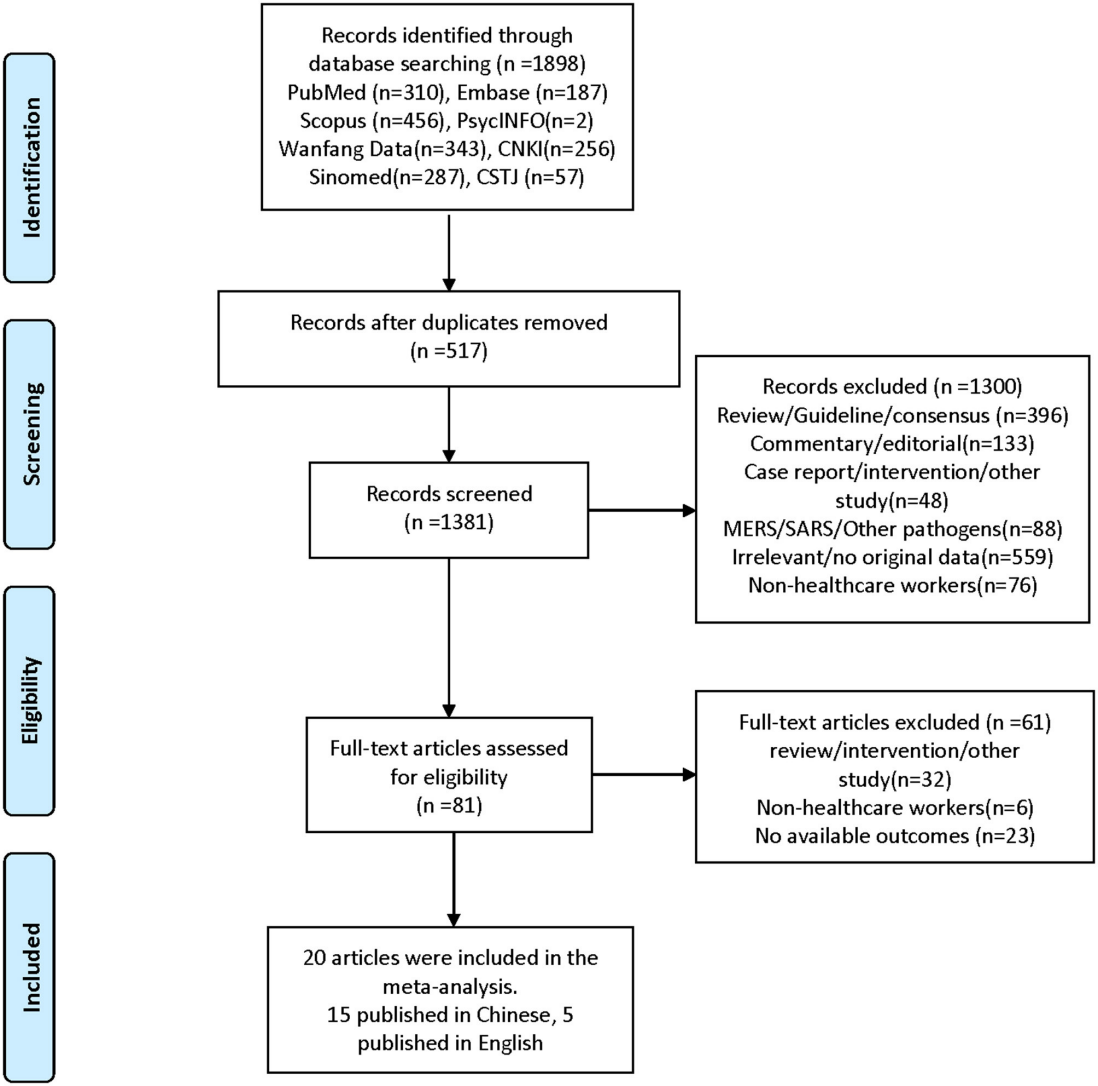
Preferred reporting items for systematic reviews and meta-analyses flow diagram of included studies (20).

According to the search strategy, a total of 1,898 records were retrieved, and 20 records were finally included (Figure 1). All studies were of a cross-sectional design, involving a total of 10,886 HCWs in 12,788 individuals for the quantitative synthesis; 70% of all participants were women, and 80% of the research were completed by February 2020. Nineteen studies took place in China, plus one in Singapore. Five studies were published in English, and the remaining 15 studies were published in Chinese. The median of participants per study was 639 (range, 37–2,299). Various instruments were utilized. For depression, the most commonly used tools were the Zung Self-Rating Depression Scale, the nine-item Patient Health Questionnaire, the Symptom Checklist-90 (SCL-90), and the 21-item Depression, Anxiety, and Stress Scale (DASS-21). For anxiety, the Zung Self-Rating Anxiety Scale, the SCL-90, and the seven-item Generalized Anxiety Disorder were used. For insomnia, the seven-item Insomnia Severity Index and the Pittsburgh Sleep Quality Index were used. For post-traumatic stress symptoms (PTSS), the Post-traumatic Stress Disorder Self-Rating Scale (PTSD-SS), the Impact of Event Scale—Revised version (IES–R), and the PTSD Checklist—Civilian version (PCL-C) were used. Obsessive–compulsive symptoms, somatization symptoms, and phobia were assessed by the SCL-90 and the 90-item Symptom Checklist—Revised. Eleven studies included frontline HCWs only, six included both frontline and second-line HCWs, and three included second-line HCWs only. When evaluated by the AHRQ assessment criteria, one study received eight points, one received seven points, four received six points, four received five points, nine received four points, and one received three points. Most studies are of moderate quality, with methodological quality scores ranging from 4 to 7. The detailed characteristics of the included studies are shown in Table 1.

**Table 1:** Characteristics of the included studies in this systematic review and meta-analysis.

**ID**	**References**	**Country**	**Age, years (mean ± SD)/%**	**Total number**	**No. of female**	**No. of HCWs**	**Survey method**	**Population**	**Instrument**	**Start date**	**End date**	**Position**	**Sampling method**	**AHRQ checklist**
1	Cai and Qin (27)	China	32.48 ± 2.03	48	37	48	Unknown	Hospital-Based	SCL-90	Unknown	7-Feb	First line	Unknown	4 yes
2	Cao et al. (28)	China	32.8 ± 9.6	37	29	37	Unknown	Hospital-Based	PHQ-9	Unknown	26-Feb	First line	Cluster	4 yes
3	Duan et al. (29)	China	32.82 ± 6.41	642	506	530	Online survey	Hospital-Based	PHQ-9, GAD-7	14-Feb	16-Feb	Mixed	Unknown	4 yes
4	He et al. (30)	China	38.7 ± 6.3	360	141	256	Online survey	Population-Based	PSQI	24-Jan	2-Mar	First line	Unknown	4 yes
5	Huang et al. (31)	China	32.6 ± 6.2	230	187	230	Online survey	Hospital-Based	SAS, PTSD-SS	7-Feb	14-Feb	First line	Cluster	4 yes
6	Iiu et al. (32)	China	29.00 ± 5.88	1,097	1,078	1,097	Online survey	Hospital-Based	PHQ-9, GAD-7, ISI-7, SQR-20	1-Feb	18-Feb	Second line	Unknown	5 yes
7	Lai et al. (15)	China	<40 (80.5%)	1,257	964	1,257	Unknown	Hospital-Based	PHQ-9, GAD-7, ISI-7, IES-R	29-Jan	3-Feb	Mixed	Cluster	7 yes
8	Li et al. (33)	China	>30 (46.6%)	205	175	205	Online survey	Hospital-Based	PCL-C	8-Feb	11-Feb	First line	Convenience	6 yes
9	Lu et al. (17)	China	<40 (78%)	2,299	1,785	2,042	Unknown	Hospital-Based	HAMD, HAMA	25-Feb	26-Feb	Mixed	Unknown	6 yes
10	Qi et al. (34)	China	≤ 40 (79%)	400	295	400	Unknown	Hospital-Based	SDS, SAS	Unknown	5-Feb	First line	Convenience	4 yes
11	Sun et al. (35)	China	<40 (97.3%)	110	102	110	Unknown	Hospital-Based	SCL-90	Unknown	25-Feb	First line	Unknown	3 yes
12	Tan et al. (36)	Singapore	31 (32, 34–41)	470	321	296	Unknown	Hospital-Based	DASS-21, IES-R	19-Feb	13-Mar	First line	Unknown	6 yes
13	Tang et al. (16)	China	33.6 ± 6.39	44	34	44	Unknown	Hospital-Based	SDS, SAS, PSS-10	Unknown	Unknown	First line	Convenience	4 yes
14	Wu et al. (42)	China	30.84 ± 4.52	106	85	106	Online survey	Hospital-Based	SAS, PSQI	Unknown	2-Feb	First line	Convenience	5 yes
15	Xiao et al. (41)	China	<40 (84.3%)	423	293	423	Online survey	Hospital-Based	SDS, SAS	6-Feb	8-Feb	Second line	Random	5 yes
16	Xu and Zhang (40)	China	31.28 ± 2.53	41	37	41	Online survey	Hospital-Based	SCL-90	Unknown	29-Jan	First line	Cluster	4 yes
17	Xu et al. (39)	China	34.79 ± 7.14	360	291	360	Online survey	Hospital-Based	SDS, SAS	7-Feb	15-Feb	Second line	Unknown	5 yes
18	Ye et al. (43)	China	≤ 35 (67.8%)	2,104	1,644	2,104	Online survey	Hospital-Based	GAD-7	29-Jan	5-Feb	Mixed	Convenience	6 yes
19	Zhang et al. (38)	China	18–60 (96.3%)	2,182	678	927	Online survey	Population-Based	PHQ-2, GAD-2, ISI-7, SCL-90-R	19-Feb	6-Mar	Mixed	Unknown	8 yes
20	Zheng et al. (37)	China	<46 (87.5%)	373	278	373	Online survey	Hospital-Based	PHQ-9	18-Feb	21-Feb	Mixed	RS	4 yes

*HCWs, healthcare workers; PHQ-9, nine-item Patient Health Questionnaire; DASS-21, 21-item Depression, Anxiety, and Stress Scale; SDS, Zung Self-Rating Depression Scale; HAMD, Hamilton Depression Scale; GAD-7, seven-item Generalized Anxiety Disorder; SAS, Zung Self-Rating Anxiety Scale; HAMA, Hamilton Anxiety Scale; ISI-7, seven-item Insomnia Severity Index; PSQI, Pittsburgh Sleep Quality Index; SQR-20, Self-Reporting Questionnaires; IES-R, Impact of Event Scale—Revised; PCL-C, PTSD Checklist—Civilian Version; PTSD-SS, Post-traumatic Stress Disorder Self-Rating Scale; SCL-90, Symptom Checklist 90; SCL-90-R, Symptom Checklist 90—Revised; AHRQ Checklist, The Agency for Healthcare Research and Quality Methodology Checklist*.

### Prevalence of Mental Health Problems in HCWs

#### Prevalence of Depressive Symptom

**Figure 2:**
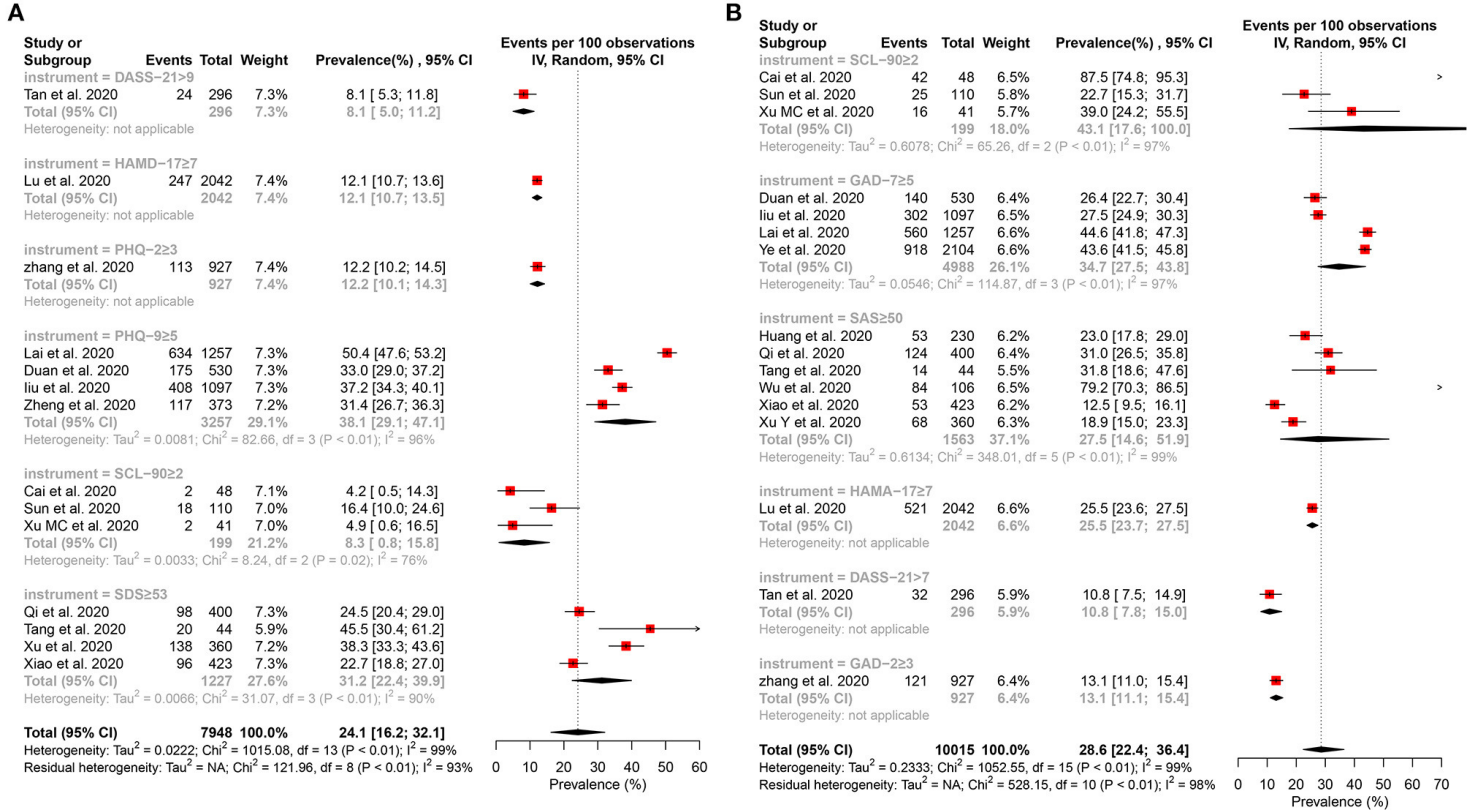
Forest plot for the meta-analysis of the prevalence of depression and anxiety among healthcare workers. **(A)** Forest plot of the prevalence of depression. **(B)** Forest plot of the prevalence of anxiety.

**Figure 3:**
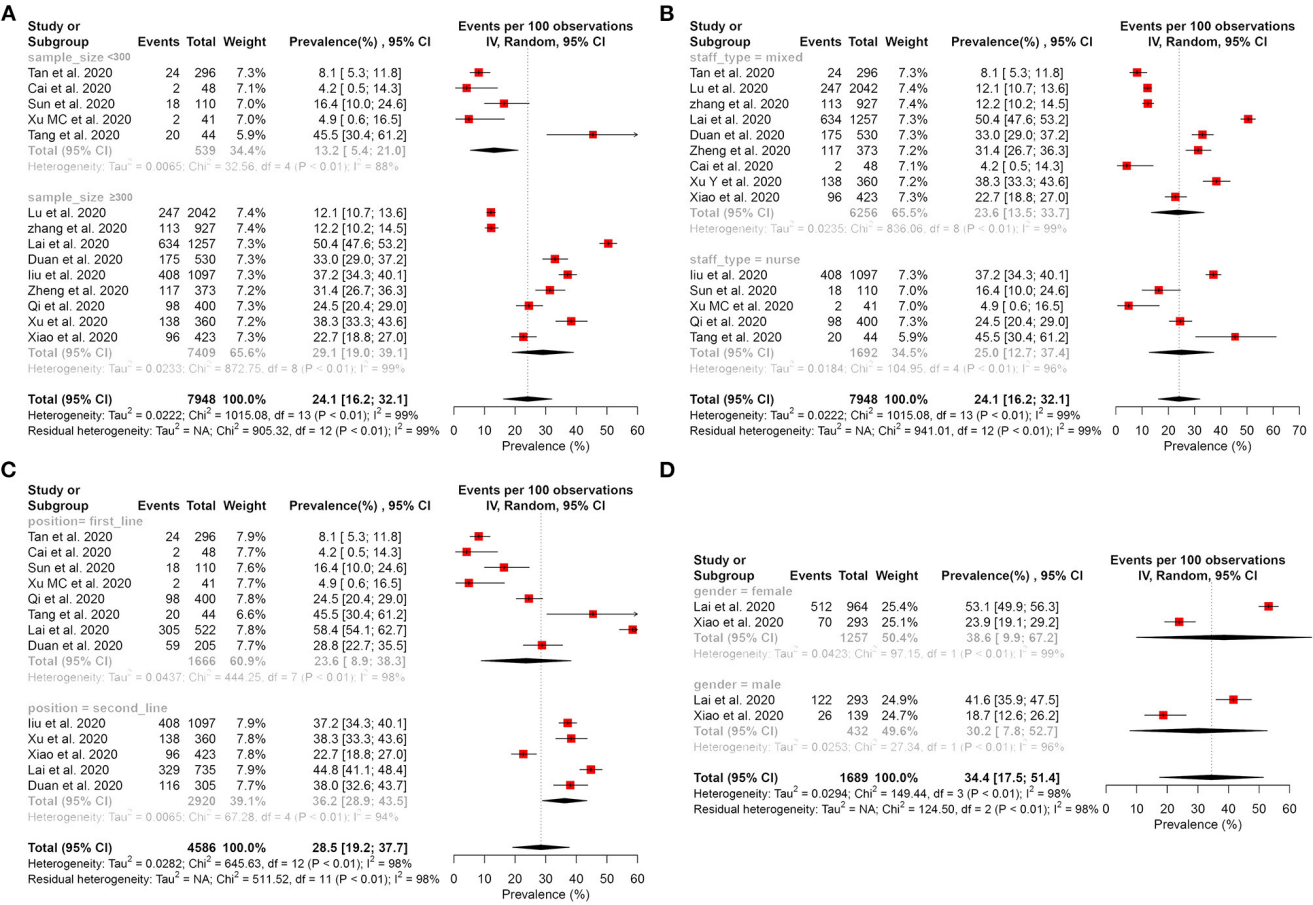
Subgroup analysis of the prevalence of depression among healthcare workers. **(A)** Subgroup analysis stratification by sample size. **(B)** Subgroup analysis stratification by staff type. **(C)** Subgroup analysis stratification by position. **(D)** Subgroup analysis stratification by gender.

**Figure 4:**
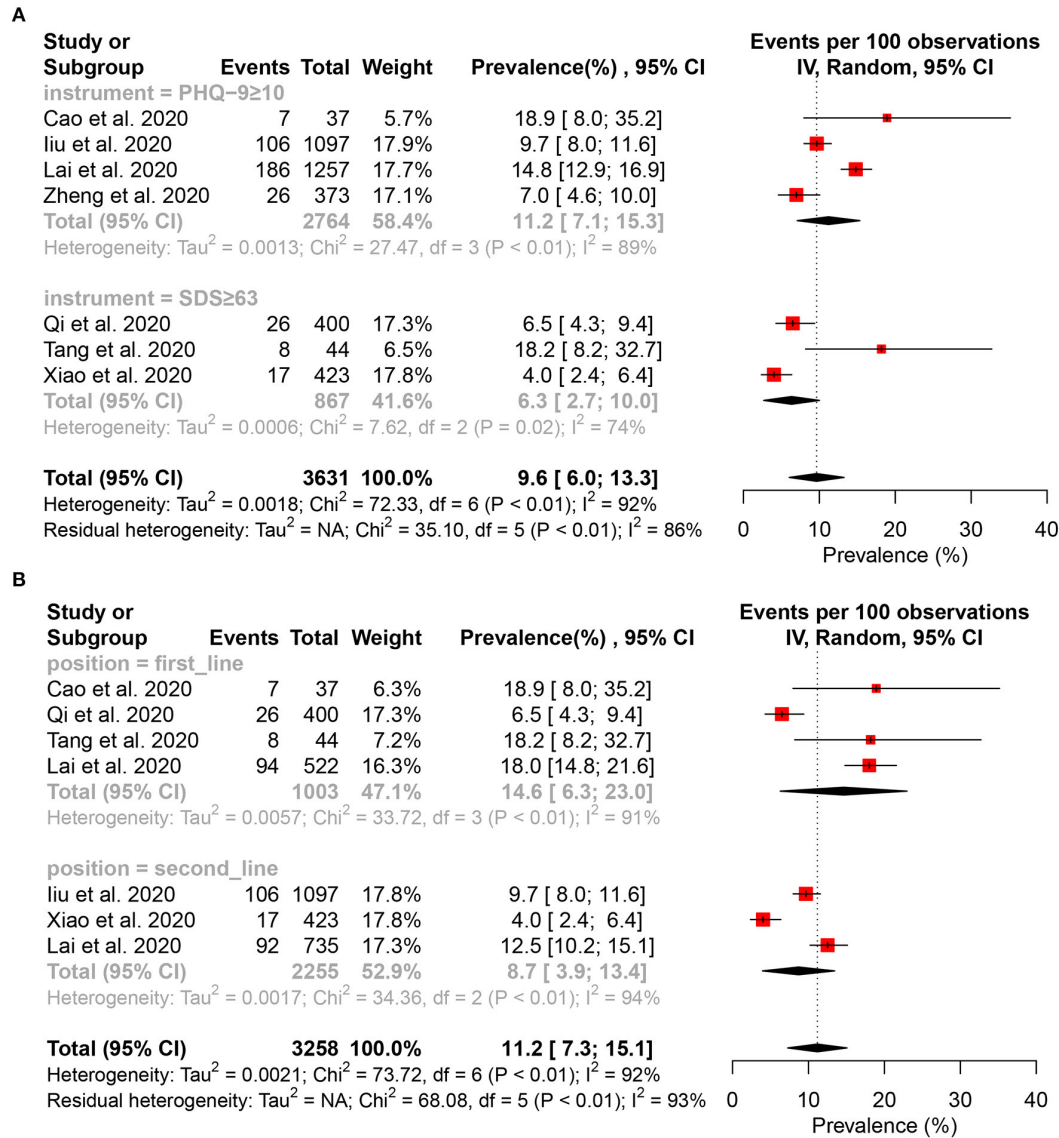
Forest plot for the meta-analysis of the prevalence of moderate to severe depression and subgroup analysis among healthcare workers. **(A)** Forest plot of the prevalence of moderate to severe depression. **(B)** Forest plot of moderate to severe depression stratification by position.

Fourteen studies (15–17, 27–29, 32, 34–41) reported that the pooled prevalence of depressive symptom among HCWs was 24.1% (95% CI: 16.2–32.1%, *I*^2^ = 99%, *P* < 0.01), with a range from 4.2 to 50.4% (Figure 2A). A subgroup analysis revealed that the second-line HCWs (36.2%, 95% CI: 28.9–43.5%, *I*^2^ = 94%, *P* < 0.01) and female HCWs (38.6%, 95% CI: 9.9–67.2%, *I*^2^ = 99%, *P* < 0.01) had a higher prevalence of depression symptom than frontline and male HCWs separately (Figure 3). About 9.6% of HCWs were identified by instruments as individuals with moderate to severe depression (Figure 4A). Among them, the prevalence of moderate to severe depression in frontline HCWs (14.6%, 95% CI: 6.3–23.0%, *I*^2^ = 91%, *P* < 0.01) is higher than those in the second-line (8.7%, 95% CI: 3.9–13.4%, *I*^2^ = 94%, *P* < 0.01; Figure 4B).

#### Prevalence of Anxiety Symptom

**Figure 5:**
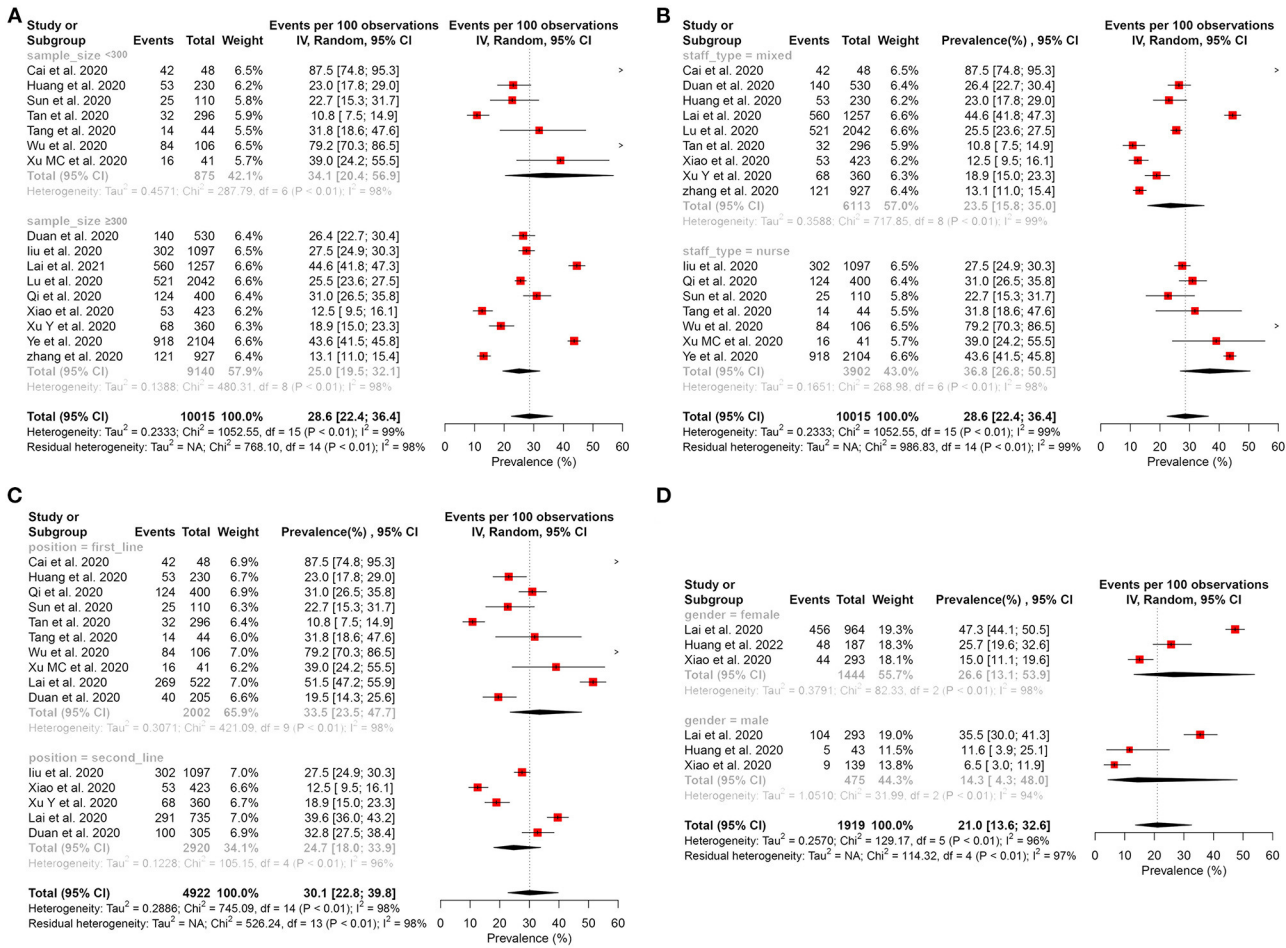
Subgroup analysis of the prevalence of anxiety among healthcare workers. **(A)** Subgroup analysis stratification by sample size. **(B)** Subgroup analysis stratification by staff type. **(C)** Subgroup analysis stratification by position. **(D)** Subgroup analysis stratification by gender.

**Figure 6:**
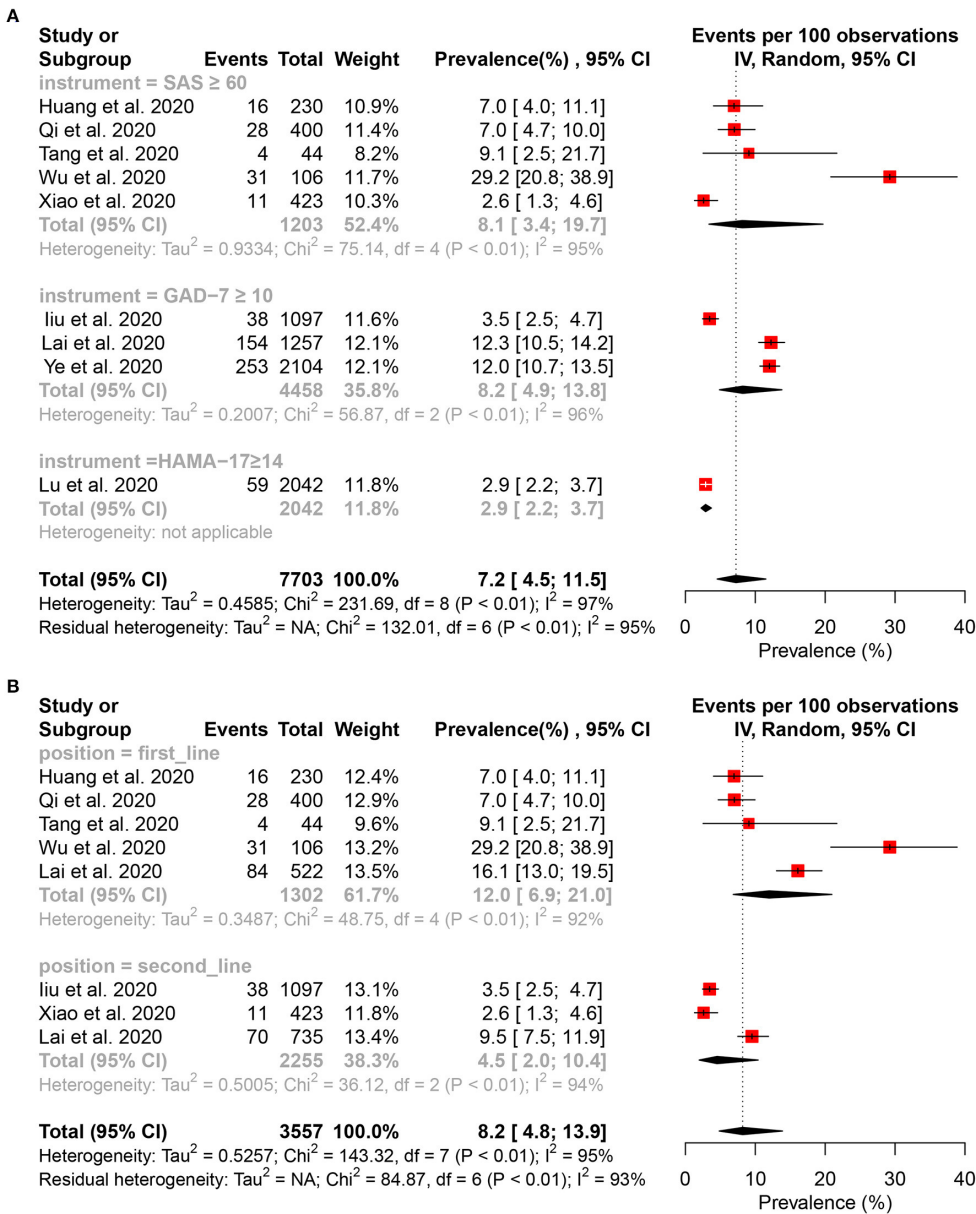
Forest plot for the meta-analysis of the prevalence of moderate to severe anxiety and subgroup analysis among healthcare workers. **(A)** Forest plot of the prevalence of moderate to severe anxiety. **(B)** Forest plot of moderate to severe anxiety stratification by position.

Sixteen studies (15–17, 27, 29, 31, 32, 34–36, 38–43) reported that the pooled prevalence of anxiety among HCWs was 28.6% (95% CI: 22.4–36.4%, *I*^2^ = 99%, *P* < 0.01), with a range from 10.8 to 87.5% (Figure 2B). In the subgroup analysis stratified by position, the frontline HCWs (33.5%, 95% CI: 23.5–47.7%, *I*^2^ = 98%, *P* < 0.01) had a higher prevalence of anxiety than the second-line HCWs (Figure 5C). Of the 16 studies, seven studies reported that the prevalence of anxiety is higher in nurses (36.8%, 95% CI: 26.8–50.5, *P* < 0.001) than that in the mixed staff type including nurses and doctors (Figure 5B). In the subgroup stratified by gender, female HCWs (26.6%, 95% CI: 13.1–53.9%, *I*^2^ = 98%, *P* < 0.01) had a higher prevalence of anxiety than male HCWs (Figure 5D). About 7.2% of HCWs were identified by instruments as individuals with moderate to severe anxiety (Figure 6A). Similar to the symptoms of depression, the anxiety symptoms of frontline HCWs are more severe than that of the second-line HCWs (Figure 6B).

#### Prevalence of Insomnia

**Figure 7:**
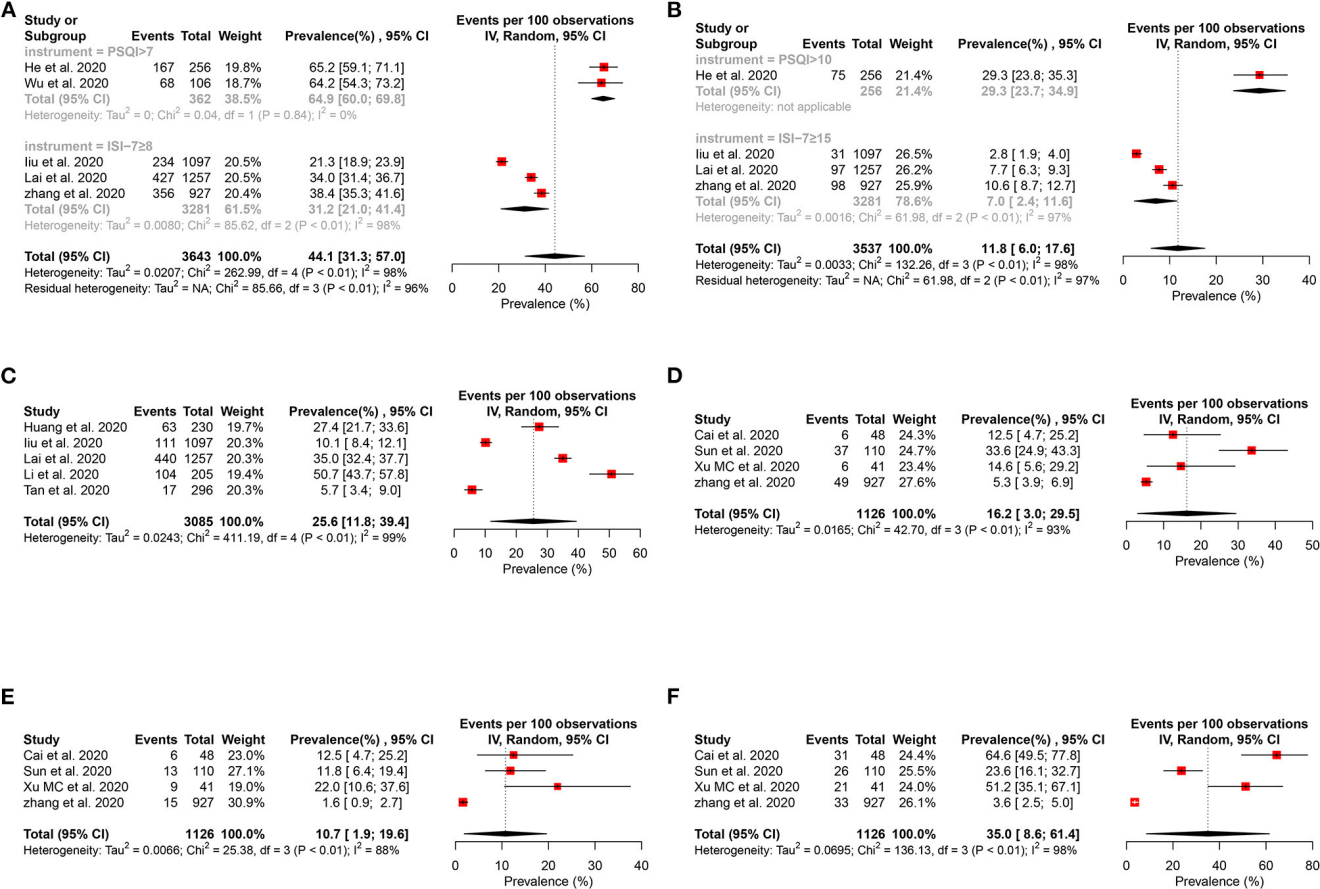
Forest plot for the meta-analysis of the prevalence of other mental health problems among healthcare workers. **(A)** Forest plot of the prevalence of insomnia. **(B)** Forest plot of the moderate to severe insomnia. **(C)** Forest plot of the prevalence of distress. **(D)** Forest plot of the prevalence of obsessive–compulsive symptoms. **(E)** Forest plot of the prevalence of somatization symptoms. **(F)** Forest plot of the prevalence of phobia.

Five studies (15, 30, 32, 38, 42) reported that the pooled prevalence of insomnia among HCWs was 44.1% (95% CI: 31.3–57.0%, *I*^2^ = 98%, *P* < 0.01), with a range from 21.3 to 65.2% (Figure 7A). About 11.8% of the HCWs were identified by instruments to be with moderate to severe anxiety (Figure 7B).

#### Prevalence of PTSS

Five studies (15, 31–33, 36) reported that the pooled prevalence of PTSS among the HCWs was 25.6% (95% CI: 11.8–39.4%, *I*^2^ = 99%, *P* < 0.01), with a range from 5.7 to 50.7% (Figure 7C).

#### Other Mental Health Problems

Four studies (27, 35, 38, 40) evaluated obsessive–compulsive symptoms (Figure 7D), somatization symptoms (Figure 7E), and phobia (Figure 7F), and their prevalence were 16.2% (95% CI: 3.0–29.5%, *I*^2^ = 93%, *P* < 0.01), 10.7% (95% CI: 1.9–19.6%, *I*^2^ = 88%, *P* < 0.01), and 35.0% (95% CI: 8.6–61.4%, *I*^2^ = 98%, *P* < 0.01) separately.

### Risk Factors of Mental Health Problems

A total of four studies reported on the risk factors of mental health problems (15, 17, 29, 38). Due to a lack of consistency in methods, outcome metrics, and control groups, a narrative synthesis of risk factors was conducted, with the main findings tabulated (Table 2). Poor health status/organic diseases, female, working in a secondary hospital, intermediate technical title, and frontline/high-risk contact with COVID-19 were the risk factors for depression. Compared with non-medical staff working in hospitals, the occupational attributes of medical staff were a protective factor. For anxiety, the risk factors were as follows: fear of COVID-19 infection, poor health status/organic diseases, female, working in a secondary hospital, intermediate technical title, frontline/high-risk contact with COVID-19, and living in rural areas. Similar to the protective factor of depression, the professional attribute of medical staff was the protective factor relative to non-medical staff working in hospitals. Working in frontline, living in rural areas, contact with COVID-19 patients, and organic diseases were the risk factors of insomnia. Female, intermediate technical title, and frontline were the risk factors of PTSS, while working outside Hubei province was the protective factor. Living in rural areas, organic diseases, and contact with patients with COVID-19 were the risk factors of obsessive–compulsive symptoms. The risk factors for somatization symptoms were living in rural areas and organic diseases.

**Table 2:** Risk factors of mental health problems among healthcare workers during the COVID-19 pandemic.

**References**	**No. of HCWs**	**Method**	**Effects**	**Risk factors for depression**	**Risk factors for anxiety**	**Risk factors for insomnia**	**Risk factors for distress**	**Risk factors for obsessive–compulsive symptoms**	**Risk factors for somatization symptoms**
Duan et al. (29)	530	Multivariable logistic regression analysis	Unadjusted OR	Poor health status, 3.16 (2.03–4.91), *p* < 0.001 Frontline medical staff, 0.37 (0.25–0.7), *p* = 0.001 (comparison: non-medical staff in the hospital) General medical staff, 0.42 (0.31–0.79), *p* = 0.003 (comparison: non-medical staff in the hospital)	Worrying about covid-19 infection, 1.86 (1.59–2.17), *p* < 0.001 Poor health status, 2.84 (1.85–4.36), *p* < 0.001 Frontline medical staff, 0.37 (0.21–0.64), *p* = 0.005 (comparison: non-medical staff in the hospital) General medical staff, 0.59 (0.36–0.95), *p* = 0.031 (comparison: non-medical staff in the hospital)	None	None	None	None
Lai et al. (15)	1257	Multivariable logistic regression analysis	Adjusted OR	Female, 1.94 (1.26–2.98), *p* = 0.003 Secondary hospital, 1.65 (1.17–2.34), *p* = 0.004 Intermediate technical title, 1.77 (1.25–2.49), *p* = 0.001(comparison: junior technical title) Frontline, 1.52 (1.11–2.09), *p* 0.01	Female, 1.69 (1.23–2.33), *p* = 0.001 Secondary hospital, 1.43 (1.08–1.90), *p* = 0.01 Intermediate technical title, 1.82 (1.38–2.39), *p* < 0.001 (comparison: junior technical title) Frontline, 1.57 (1.22–2.02), *p* < 0.001	Frontline, 2.97 (1.92–4.60), *p* < 0.001	Female, 1.45 (1.08–1.96), *p* = 0.01 Intermediate technical title, 1.94 (1.48–2.55), *p* < 0.001 (comparison: junior technical title) Frontline, 1.60 (1.25–2.04), *p* < 0.001 Outside Hubei province, 0.62 (0.43–0.88), *p* = 0.008	None	None
Lu et al. (17)	2042	Ordinal logistic regression model	Unadjusted OR	High-risk contact, 2.016 (1.102–3.685), *p* = 0.023	High-risk contact, 2.062 (1.349–3.153), *p* = 0.001	None	None	None	None
Zhang et al. (38)	927	Multivariable logistic regression analysis	Unadjusted OR	Female, 1.85 (1.11–3.08), 0.02 Organic diseases, 2.51 (1.51–4.18), *p* < 0.01	Female, 1.80 (1.10–2.95), *p* = 0.02 Living in rural areas, 1.88 (1.09–3.21), *p* = 0.02 Contact with COVID-19 patients, 2.06 (1.28–3.32), *p* < 0.01 Organic diseases, 2.85 (1.73–4.68), *p* < 0.01	Living in rural areas, 2.18 (1.42–3.35), *p* < 0.01 Contact with COVID-19 patients, 2.53 (1.74–3.68), *p* < 0.01 Organic diseases, 3.39 (2.20–5.22), *p* < 0.01	None	Living in rural areas, 2.49 (1.21, 5.11), *p* = 0.01 Contact with COVID-19 patients, 3.27 (1.75–6.11), *p* < 0.01 Organic diseases, 2.24 (1.07–4.71), *p* = 0.03	Living in rural areas, 4.78 (1.55–14.76), *p* < 0.01 Organic diseases, 7.89 (2.75–22.62), *p* < 0.01

### Heterogeneity Analysis

To identify potential sources of heterogeneity, a subgroup analysis was conducted. However, high heterogeneity was not significantly explained by sample size, staff type, position, and gender (Figures 3, 5). In the univariate meta-regression analyses of the prevalence of depression, a significant estimate was found for the covariate of instruments with *R*^2^ (amount of heterogeneity accounted for) = 67.75%, *P* < 0.0001. No significant estimates were found for the covariates of sample size (less or more than 300), hospital (survey in one or more hospital), country (China or another country), position (frontline or second-line), or staff type (nurses or mixed with nurses and doctors). The meta-regression showed that country was significantly associated with the prevalence of anxiety (*R*^2^ = 4.63%, *P* < 0.0001); however, it was not significantly correlated with instrument (*R*^2^ = 18.11%, *P* = 0.0533), sample size (*R*^2^ = 19.56%, *P* = 0.1469), hospital (*R*^2^ = 0.00%, *P* = 0.7880), position (*R*^2^ = 11.11%, *P* = 0.1744), and staff type (*R*^2^ = 0.00%, *P* = 0.1030). A sensitivity analysis was conducted by excluding, one by one, the included studies that demonstrated no substantial alteration (Supplementary Tables 1, 2: Supplementary Figures 1, 2).

### Publication Bias

The funnel plot for the primary outcomes seems somewhat asymmetrical (Supplementary Figures 3, 4). However, the Egger's linear regression test of funnel plot asymmetry was performed, and it indicated no significant asymmetry (*P*_depression_ = 0.3001, *P*_anxiety_ = 0.1045).

## Discussion

The present study investigates the prevalence and risk factors of mental health problems among HCWs during the COVID-19 pandemic based on 10,886 HCWs summarized in 20 cross-sectional studies. According to our research, the prevalence of depression, anxiety, insomnia, PTSS, phobia, obsessive–compulsive symptoms, and somatization symptoms was 24.1, 28.6, 44.1, 25.6, 35.0, 16.2, and 10.7%, respectively. These findings highlight an important issue in HCWs during the COVID-19 pandemic.

It is no surprise that HCWs have a much higher prevalence of mental health problems during the COVID-19 pandemic. There are many factors that can explain this. The ever-increasing number of confirmed and suspected cases, overwhelming workload, depletion of personal protection equipment, widespread media coverage, lack of specific drugs, and feelings of being inadequately supported may all contribute to the mental burden of these HCWs (44). Previous studies showed that HCWs feared contagion and infection of their family and experienced high levels of PTSS, anxiety, and depression symptoms during the outbreak of SARS in 2003 (45, 46). The mental health problems faced by medical staff may be related to many difficulties in work safety, such as the insufficient understanding of the disease at the initial stage, the lack of knowledge concerning prevention and control, the long-term heavy workload, the high risk of exposure to confirmed or suspected cases, the shortage of medical protective equipment, the lack of rest, and the exposure to critical life events during the COVID-19 pandemic (38, 47).

It is worth noting that 25.6% of HCWs suffer from PTSS. Post-traumatic stress disorder (PTSD) is a common consequence of major disasters. During the COVID-19 pandemic, HCWs have endured huge threats and unprecedented challenges, which may cause them to develop acute stress disorder that will potentially degenerate into chronic PTSD over time. A survey conducted 2 months after the outbreak of SARS in Singapore revealed that ~20% of HCWs were suffering from PTSD (48). What is more, a cohort study that lasted 30 months post-SARS among SARS survivors found that HCWs have a much higher percentage of chronic PTSD than non-HCWs (40.7 vs. 19%; *P* = 0.031) (49). Additionally, female, working in frontline, and intermediate technical title were the risk factors of PTSS during the COVID-19 pandemic; however, working outside Hubei province was the protective factor (15).

We found that 70% of all participants were female (most of whom were nurses). Moreover, a subgroup analysis revealed that females and nurses had a high prevalence of depression and anxiety. During the SARS outbreak, a study conducted among HCWs in emergency departments also showed that nurses were more likely to develop distress than physicians (50). During the COVID-19 pandemic, frontline nurses may be at risk of infection due to the close and frequent contact with patients and the longer-than-usual working hours during the COVID-19 pandemic. This also reminds us that the society should be more concerned on the mental health of women and nurses during the major epidemic.

Another important finding in the subgroup analysis revealed that the frontline HCWs had a higher prevalence of anxiety and a lower prevalence of depression than the second-line HCWs. A high level of anxiety in the early stage of the emerging infectious disease may be an adaptive defense mechanism response to potentially threatening events (51). However, when it is chronic or disproportionate, it becomes harmful and can be a key component in the development of various psychiatric disorders (51, 52). What deserves our attention is that, compared with the second-line HCWs, the proportions of moderate-to-severe anxiety and depression are higher among the frontline staff. Working in the center of a pandemic area such as Wuhan or high-risk contact with COVID-19 patients in frontline positions, such as the emergency department, respiratory department, fever clinic, etc., is a risk factor for mental health problems (15, 17, 38). For this reason, we should pay more attention to the frontline medical staff. Timely screening and appropriate intervention are important to reduce the severity and chronicity of mental health problems.

In addition, the physical condition of medical staff was an important risk factor for mental health problems. The prevalence of depression, anxiety, insomnia, obsessive–compulsive symptoms, and somatization symptoms of medical staff with poor health conditions or comorbidities of organic diseases is higher than that of the healthy ones (29, 38). Surprisingly, living in rural areas is a risk factor for anxiety, insomnia, obsessive–compulsive symptoms, and somatization symptoms (38). Differences in the working environment, medical technology, and the knowledge of COVID-19 may partially explain this phenomenon.

Limitations should be considered when interpreting the findings of this study. First, it was limited in scope. Of the 20 studies, 19 are from China, 15 of which are published in Chinese and thus limiting the generalization of other countries. Second, it is important to note that the vast majority of participants were assessed by a self-rating scale rather than by gold-standard diagnostic clinical interviews for mental health disorders, and the duration of symptoms of most participants did not meet the diagnostic criteria. The sensitivity and the specificity of these instruments for diagnosing mental health problems vary substantially. Third, all studies are cross-sectional studies, and there is no longitudinal study. Moreover, most of them were completed in February 2020 or earlier. With the increasingly arduous situation, the mental health symptoms of HCWs could become more severe. Fourth, many other factors that could predispose medical staff to anxiety, for example, family history and emotional trauma, could not be assessed due to the wide variability of factors examined in the studies. Finally, only a few studies have explored the risk factors of mental health problems, which are not sufficient to fully understand the problem. Moreover, all studies on risk factors were of a cross-sectional design, without baseline control and follow-up data, so it is impossible to determine the causal relationship between them. Some risk factor studies have not controlled for confounding factors and cannot exclude the influence of factors such as working position, COVID-19 exposure intensity, and some sociodemographic factors.

## Conclusions

In this systematic review, HCWs have a relatively high prevalence of depression, anxiety, insomnia, PTSS, phobia, obsessive–compulsive symptoms, and somatization symptoms during the COVID-19 pandemic, and focus should be on the HCWs at high risk of mental health problems. Further research is needed to identify effective strategies for preventing and treating mental health problems among HCWs during the COVID-19 pandemic.

## Data Availability Statement

The raw data supporting the conclusions of this article will be made available by the authors, without undue reservation.

## Author Contributions

QH, DW, HW, and QW contributed to research conception and study design and contributed to data analysis/interpretation. QH, DW, MX, YT, YD, LZ, MD, and YW contributed to search and data acquisition. QH and DW contributed to statistical analysis and manuscript writing. QW and HW took responsibility that this study has been reported honestly, accurately, transparently, and contributed very important intellectual content during manuscript drafting or revision and accepts accountability for the work. All authors read and approved the final manuscript.

## Conflict of Interest

The authors declare that the research was conducted in the absence of any commercial or financial relationships that could be construed as a potential conflict of interest.
