# Supplementary material for: Prevalence and Risk Factors of Mental Health Problems Among Healthcare Workers During the COVID-19 Pandemic: A Systematic Review and Meta-Analysis
Source: Front Psychiatry. 2021 Jun 15;12:567381. doi: 10.3389/fpsyt.2021.567381 (PMC8239157; doi:10.3389/fpsyt.2021.567381)
Supplement: Supplementary file 1 [file Data_Sheet_1.pdf]

Prevalence and risk factors of mental health problems among healthcare workers during the COVID-19 pandemic: a systematic review and meta-analysis

Qinjian Hao[1a], Dahai Wang[2a], Min Xie[3], Yiguo Tang[3], Yikai Dou[3], Ling Zhu[3], Yulu Wu[3], Minhan Dai[3], Hongmei Wu[1*], Qiang Wang[3*]

Item S1：Detailed search strategies for each database.

PubMed search strategy

#1. "coronavirus"[MeSH Terms] OR "coronavirus"[All Fields] OR "coronaviruses"[All Fields] OR "corona virus"[All Fields] OR "severe acute respiratory syndrome coronavirus 2"[Supplementary Concept] OR "severe acute respiratory syndrome coronavirus 2"[All Fields] OR "SARS-CoV-2"[All Fields] OR "2019-ncov"[All Fields] OR "COVID-19"[All Fields] OR "COVID-2019"[All Fields]

#2. "mental health"[MeSH Terms] OR "mental"[All Fields] OR "anxiety disorders"[MeSH Terms] OR "anxiet*"[All Fields] OR "phobic disorders"[MeSH Terms] OR "phobic"[All Fields] OR "phobia"[All Fields] OR "panic disorder"[MeSH Terms] OR "panic"[All Fields] OR "mood disorders"[MeSH Terms] OR "mood"[All Fields] OR "emotion*"[All Fields] OR "depressive disorder"[MeSH Terms] OR "depress*"[All Fields] OR "Stress Disorders, Traumatic"[MeSH Terms] OR "PTSD"[All Fields] OR "stress*"[All Fields] OR "sleep wake disorders"[MeSH Terms] OR "sleep*"[All Fields] OR "insomnia"[All Fields] OR "dyssomnia"[All Fields] OR "psych*"[All Fields]

#3. 2020/1/1:3000/12/31[Date - Publication]

#4 #1 AND #2 AND #3

Embase search strategy

#1. 'coronavirus'/exp OR 'coronavirus' OR 'coronaviruses' OR 'corona virus'/exp OR 'corona virus' OR '2019-ncov' OR 'covid-19' OR 'covid-2019' OR 'sars-cov-2'

#2. 'mental health'/exp OR 'mental' OR 'anxiety disorder'/exp OR 'anxiet*' OR 'phobia'/exp OR 'phobic' OR 'phobia' OR 'panic'/exp OR 'panic' OR 'mood disorder'/exp OR 'mood' OR 'emotio*' OR 'depression'/exp OR 'depress*' OR 'stress'/exp OR 'posttraumatic stress disorder'/exp OR 'acute stress disorder'/exp OR 'stress' OR 'sleep disorder'/exp OR 'sleep' OR 'insomnia' OR 'dyssomnia' OR 'psych*'

#3. #1 AN #3

#4. #3 AND [2020-2020]/py

Scopus search strategy

#1. (ALL (coronavirus) OR ALL (coronaviruses) OR ALL ("corona virus") OR ALL ("severe acute respiratory syndrome coronavirus 2") OR ALL (sars-cov-2) OR ALL (2019-ncov) OR ALL (covid-19) OR ALL (covid-2019))

#2. (ALL (mental) OR ALL (anxiet*) OR ALL (phobic) OR ALL (phobia) OR ALL (panic) OR ALL (mood) OR ALL (emotion*) OR ALL (depress*) OR ALL (stress*) OR ALL (sleep*) OR ALL (insomnia) OR ALL (dyssomnia) OR ALL (psych*))

#3. #1 AND #2

#4. #3 AND (LIMIT-TO (PUBYEAR , 2020))

PsycINFO search strategy

#1. ((Any Field: (coronavirus)) OR (Any Field: (coronaviruses)) OR (Any Field: ("corona virus")) OR (Any Field: ("severe acute respiratory syndrome coronavirus 2")) OR (Any Field: (SARS-CoV-2)) OR (Any Field: (2019-ncov)) OR (Any Field: (COVID-19)) OR (Any Field: (COVID-2019)))

#2. ((Any Field: (mental)) OR (Any Field: (anxiet*)) OR (Any Field: (phobic)) OR (Any Field: (phobia)) OR (Any Field: (panic)) OR (Any Field: (mood)) OR (Any Field: (emotion*)) OR (Any Field: (depress*)) OR (Any Field: (stress)) OR (Any Field: (sleep)) OR (Any Field: (insomnia)) OR (Any Field: (dyssomnia)) OR (Any Field: (psych*)))

#3. #1 AND #2

#4. #3 AND Year: 2020

Table S1: Influential analysis of the prevalence of depression (Random effects model)

study	proportion	95%-CI p-value	tau^2	tau	I^2
Omitting Cai et al. 2020	0.2566	[0.1742; 0.3389]	0.0222	0.1489	98.8%
Omitting Duan et al. 2020	0.2343	[0.1509; 0.3177]	0.0227	0.1507	98.8%
Omitting Iiu et al. 2020	0.2308	[0.1495; 0.3121]	0.0215	0.1467	98.6%

Omitting Lai et al. 2020	0.2189	[0.1554; 0.2824]	0.0128	0.1133	97.7%
Omitting Lu et al. 2020	0.2510	[0.1629; 0.3392]	0.0254	0.1595	98.5%
Omitting Qi et al. 2020	0.2410	[0.1564; 0.3257]	0.0234	0.1530	98.8%
Omitting Sun et al. 2020	0.2471	[0.1639; 0.3303]	0.0226	0.1505	98.8%
Omitting Tan et al. 2020	0.2539	[0.1700; 0.3378]	0.0230	0.1516	98.7%
Omitting Tang et al. 2020	0.2279	[0.1462; 0.3095]	0.0221	0.1485	98.8%
Omitting Xiao et al. 2020	0.2425	[0.1575; 0.3275]	0.0236	0.1537	98.8%
Omitting Xu MC et al. 2020	0.2558	[0.1734; 0.3383]	0.0222	0.1491	98.8%
Omitting Xu Y et al. 2020	0.2302	[0.1482; 0.3123]	0.0220	0.1482	98.8%
Omitting zhang et al. 2020	0.2509	[0.1624; 0.3394]	0.0256	0.1601	98.7%
Omitting Zheng et al. 2020	0.2356	[0.1522; 0.3191]	0.0227	0.1508	98.8%
Pooled estimate	0.2412	[0.1618; 0.3207]	0.0222	0.1489	98.7%

Figure S1: Forest plot for influential analysis of the prevalence of depression

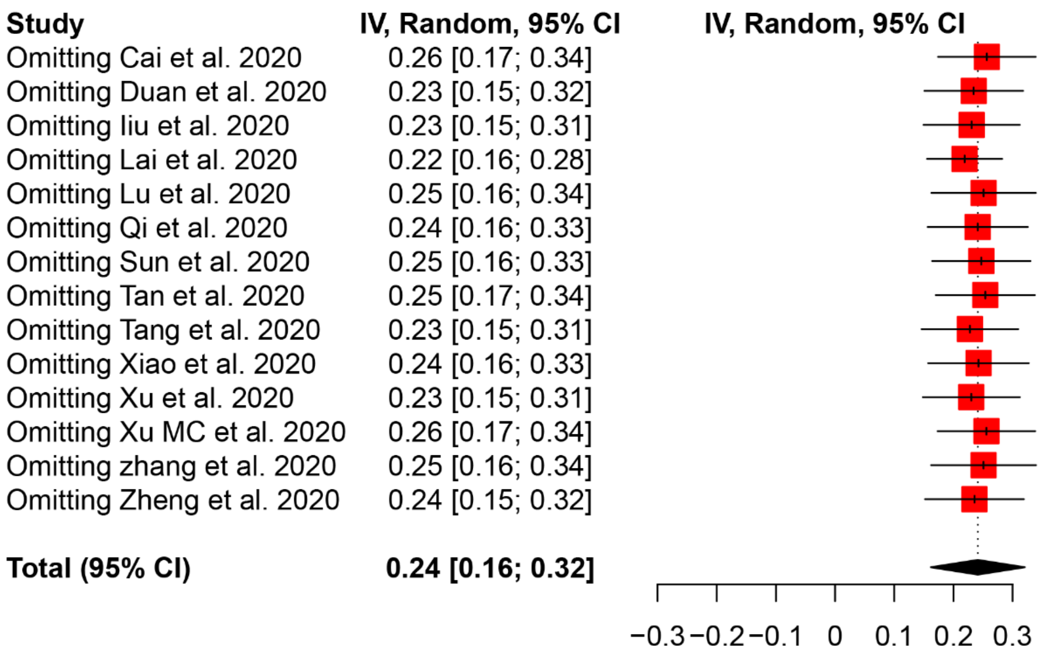

Table S2: Influential analysis of the prevalence of anxiety (Random effects model)

study	proportion	95%-CI p-value	tau^2	tau	I^2
Omitting Cai et al. 2020	0.2645	[0.2099; 0.3334]	0.1956	0.4422	98.3%
Omitting Duan et al. 2020	0.2871	[0.2227; 0.3701]	0.2388	0.4886	98.6%
Omitting Huang et al. 2020	0.2897	[0.2254; 0.3724]	0.2335	0.4832	98.6%
Omitting Iiu et al. 2020	0.2862	[0.2210; 0.3706]	0.2474	0.4974	98.6%
Omitting Lai et al. 2021	0.2763	[0.2089; 0.3656]	0.2919	0.5403	98.6%
Omitting Lu et al. 2020	0.2878	[0.2227; 0.3717]	0.2425	0.4924	98.5%
Omitting Qi et al. 2020	0.2839	[0.2198; 0.3667]	0.2427	0.4926	98.7%
Omitting Sun et al. 2020	0.2897	[0.2255; 0.3721]	0.2334	0.4831	98.7%

Omitting Tan et al. 2020	0.3036	[0.2376; 0.3878]	0.2225	0.4717	98.6%
Omitting Tang et al. 2020	0.2838	[0.2209; 0.3646]	0.2346	0.4843	98.7%
Omitting Wu et al. 2020	0.2662	[0.2105; 0.3368]	0.2024	0.4499	98.3%
Omitting Xiao et al. 2020	0.3016	[0.2364; 0.3849]	0.2196	0.4686	98.6%
Omitting Xu MC et al. 2020	0.2803	[0.2180; 0.3603]	0.2351	0.4849	98.7%
Omitting Xu Y et al. 2020	0.2937	[0.2290; 0.3766]	0.2287	0.4783	98.6%
Omitting Ye et al. 2020	0.2766	[0.2066; 0.3703]	0.3184	0.5643	98.6%
Omitting zhang et al. 2020	0.3018	[0.2385; 0.3818]	0.2032	0.4507	98.4%
Pooled estimate	0.2856	[0.2240; 0.3642]	0.2333	0.4830	98.6%

Figure S2: Forest plot for influential analysis of the prevalence of anxiety

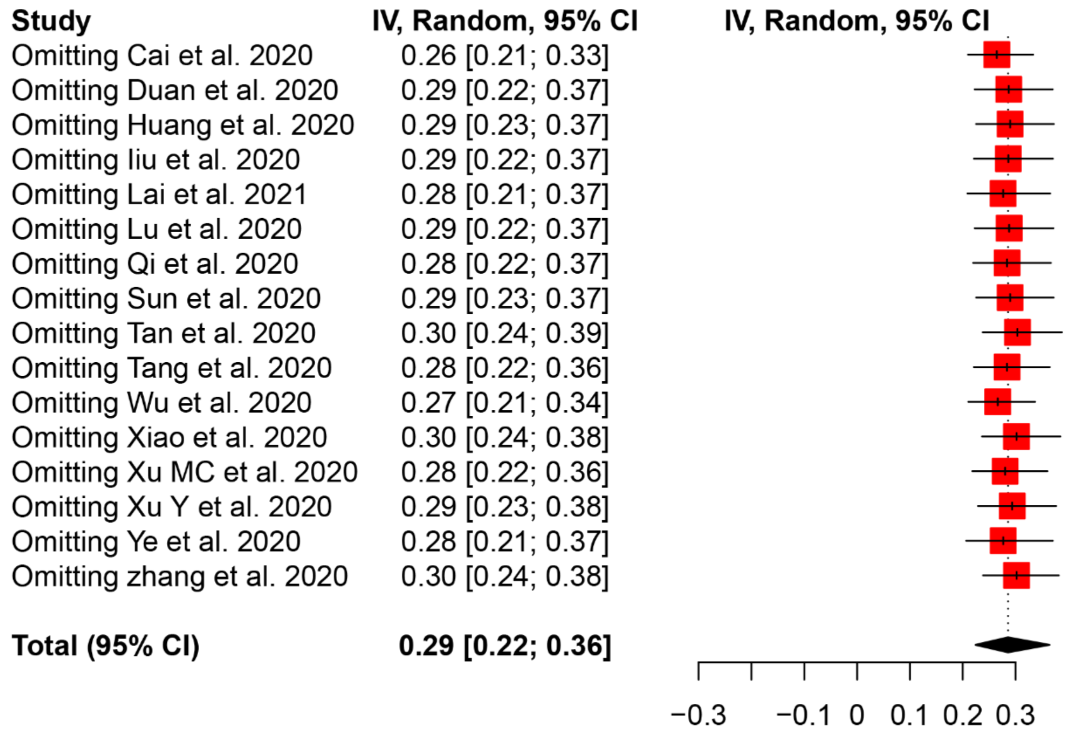

Figure S3: Funnel plot(a) and Egger's linear regression(b) for meta-analysis of the prevalence of depression

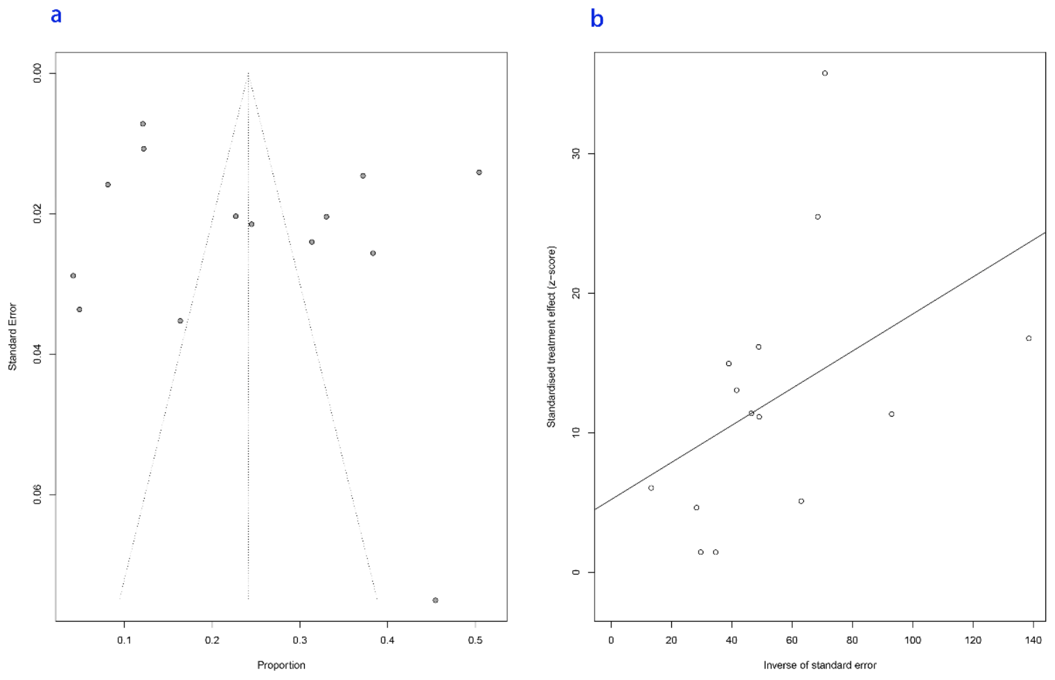

Figure S4: Funnel plot(a) and Egger's linear regression(b) for meta-analysis of the prevalence of anxiety

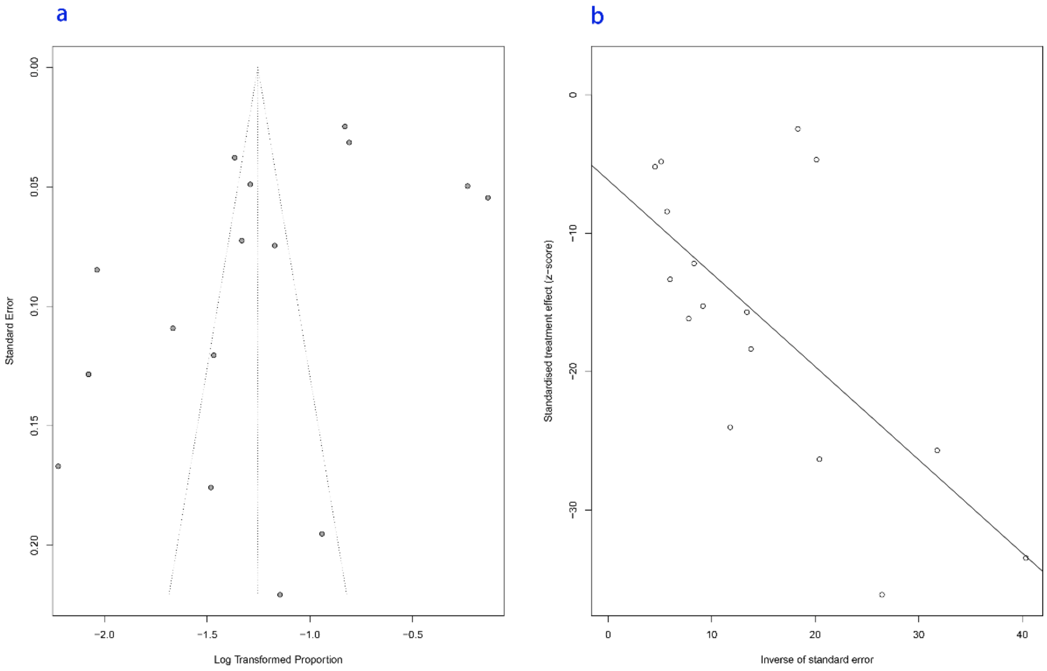
a
b
Standard Error
Log Transformed Proportion
Standardised treatment effect (z-score)
Inverse of standard error
